# A Novel Defined RAS-Related Gene Signature for Predicting the Prognosis and Characterization of Biological Function in Osteosarcoma

**DOI:** 10.1155/2022/5939158

**Published:** 2022-08-23

**Authors:** Qin Chen, Xueliang Zhou, Jianqiang Jin, Jiangbiao Feng, Zhounan Xu, Yunping Chen, Haibo Zhao, Zhongchen Li

**Affiliations:** ^1^Zhejiang Province People's Hospital Haining Hospital, Haining, China; ^2^The 903 Hospital of the Chinese People's Liberation Army, Beijing, China

## Abstract

**Background:**

Osteosarcoma (OS) is the most common primary bone malignancy in children and adolescents with a high incidence and poor prognosis. Activation of the RAS pathway promotes progression and metastasis of osteosarcoma. RAS has been studied in many different tumors; however, the prognostic value of RAS-associated genes in OS remains unclear. On this basis, we investigated the RAS-related gene signature and explored the intrinsic biological features of OS.

**Methods:**

We obtained RNA transcriptome sequencing data and clinical information of osteosarcoma patients from the TARGET database. RAS pathway-related genes were obtained from the KEGG pathway database. Molecular subgroups and risk models were developed using consensus clustering and least absolute shrinkage and selection operator (LASSO) regression, respectively. ESTIMATE algorithm and ssGSEA analysis were used to assess the tumor microenvironment and immune penetrance between the two groups. A comprehensive review of gene ontology (GO) and KEGG analyses revealed inherent biological functional differences between the two groups.

**Results:**

The consistent clustering showed stratification of osteosarcoma patients into two subtypes based on RAS-associated genes and provided a robust prediction of prognosis. A risk model further confirmed that RAS-related genes are the best prognostic indicators for OS patients. GO analysis showed that GDP/GTP binding, focal adhesion, cytoskeletal motor activity, and cell-matrix junctions were associated with the RAS-related model group. Furthermore, RAS signaling in osteosarcoma based on KEGG analysis was significantly associated with cancer progression, with immune function and tumor microenvironment particularly affected.

**Conclusion:**

We constructed a prognostic model founded on RAS-related gene and demonstrated its predictive ability. Then, furtherly exploration of the molecular mechanisms and immune characteristics proved the role of RAS-related gene in the dysregulation in OS.

## 1. Introduction

Although osteosarcoma (OS) is the most frequent type of primary bone cancer among pediatric population, it is a rare type of disease (5.6 cases per million under 15) and accounts for 2% of childhood cancers [1–3]. Osteosarcoma occurs predominantly in adolescents, which corresponds with pubertal development. But another incidence peak occurs in the elderly and is often related to Paget's disease [4]. The most common site of origin for osteosarcoma has been reported to be epiphysis [5]. If untreated, osteosarcoma has a rapid course of disease progression and >90% of patients died from pulmonary metastases [6].

Standard treatment for osteosarcoma includes surgical resection and adjuvant chemotherapy, resulting in a long-term survival rate of 65–70% [7]. However, for patients with distant metastases at initial presentation, the reported survival rate varies from only 10% to 40% [8]. Since osteosarcoma is an infrequent and aggressive tumor for which treatment has remained unchanged for decades, it is challenging to reduce therapy resistance and improve prognosis of patients with distant metastases. Hence, there is a necessity to assess the genetic features of this disease and explore novel treatment strategy.

Several genetic alterations have been described in osteosarcoma, such as *TP53* mutation and *RB1* deletions [9], which are involved in different molecular pathways associated with tumor progression. RAS-related proteins appertain to the family of GTPases, and *RAS* (*HRAS*, *NRAS*, and *KRAS*) is regarded as oncogene due to its mutations [10]. Alterations and abnormal activations in the RAS pathway are frequently linked with multitude biological events, including abnormal cell proliferation, developmental disorders, and tumorigenesis. Hyperactive RAS signaling pathway has emerged as a major tumorigenesis and is relevant with a high cancer risk in certain tumors [11].

Considering the difficulty of direct targeting of RAS proteins, indirectly inhibiting one of the RAS pathway molecules can be used in therapeutic approaches [12]. Currently, targeting RAS upstream molecule such as tyrosine kinase receptors, or downstream effectors show treatment efficacy both in vitro and in vivo and deserve further investigations in osteosarcoma patients [13–16]. Accordingly, discovery of characteristic RAS-related gene signatures in osteosarcoma may be helpful to reflect tumor heterogeneity and guide individual treatment for patients.

As of now, it is not known how RAS-related gene expression patterns differ among osteosarcoma patients. In the present study, we performed bioinformatics and statistical analyses to identify and validate a RAS-related gene signature in osteosarcoma. Besides, the biology function and intratumoral immune landscape were depicted comprehensively.

## 2. Methods

### 2.1. Collection of Datasets on Osteosarcoma

Clinical data and RNA sequencing data regarding osteosarcoma patients were acquired from the clinical data and RNA-seq data of osteosarcoma patients were obtained from the Therapeutic Research to Generate Effective Treatment (TARGET; https://ocg.cancer.gov/programs/target) database, which included a total of 84 samples. RAS pathway-related genes were retrieved through the KEGG pathway database (KEGG pathway: hsa04014 (genome.jp)).

### 2.2. Recognition of Molecular Subgroups and Time Assessment

At first, a univariate Cox regression analysis was used to identify 14 genes associated with the prognosis of osteosarcoma. Based on the expression matrix from the 14 genes, consensus clustering was performed using the *R* package “ConsensusClusterPlus.” The calculation of stromal score, immune score, and tumor purity was performed using the algorithm for estimating stromal and immune cells in malignant tissues using expression data (ESTIMATE) [17]. The extent of enrichment of 24 immune infiltrating cells in tumor samples was assessed using single-sample gene set enrichment analysis (ssGSEA) [18].

### 2.3. Identification of Differentially Expressed RAS-Related Genes

The Limma package version 3.4.3 in *R* software version 4.0.3 was used to identify DEGs by comparing the mRNA expression of RAS pathway-associated genes between tumor and normal tissues in the TARGET dataset. Those genes with FDR <0.05 and |log2 FC| >0.585 were selected for further analysis.

### 2.4. Functional Analyses

The differentially expressed genes (DEGs) between both clusters were identified using the *R* package “Limma.” The *R* package “clusterProfiler” [19] was used for gene ontology (GO) analysis and Kyoto Encyclopedia of Genes and Genomes (KEGG) analysis to enrich correlation pathways for visualization in Metascape.

### 2.5. Establishment of the RAS-Related Gene Prognostic Model

In order to assess the prognostic value of RAS-associated genes, we used additional Cox regression analysis to assess the correlation between each gene and survival status. We set the *P* value to a critical value of 0.05 to avoid omission and identified 14 survival-associated genes for further analysis. Then, LASSO Cox regression models (*R* package “glmnet”) were used to narrow down the candidate genes and build predictive models.

Finally, five genes and their coefficients were retained, and the penalty parameter (*λ*) was set according to the minimum criterion. After centralizing and normalizing the gene expression data (using the “scale” function in *R*), the risk score was calculated with the following formula: risk score = risk score =  (0.139 *∗* GNG4 index) + (−1.628 *∗* RAB5C index) + (−0.931 *∗* PRKACB index) + (0.447 *∗* EFNA5 index) + (0.288 *∗* EFNA1 index). OS patients were divided into low and high-risk subgroups according to the median risk score, and the time to OS was compared between the two subgroups by Kaplan–Meier analysis. The predictive efficiency of the model was assessed using the ROC and Martingale residual method.

### 2.6. Enrichment Analysis of the Function of DEGs between the Low and High-Risk Groups

**Figure 1:**
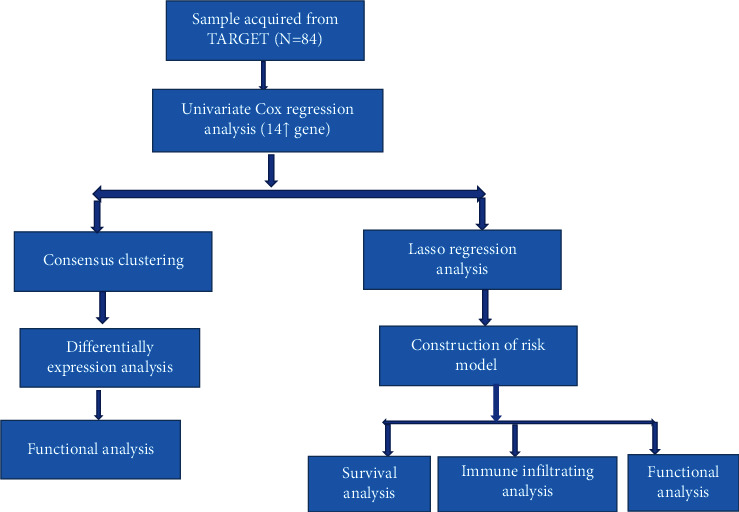
Flow diagram of the data analysis process.

The patients with OS in the TCGA cohort were stratified into two subgroups based on the median risk score. Screening of DEGs between low and high- risk groups was performed according to specific criteria (|log2FC| ≥1 and FDR<0.05). On the basis of these, DEGs, GO, and KEGG analyses were performed using the software package “clusterProfiler.” The package “gsva” [20] was used to perform ssGSEA, to calculate the fraction of infiltrating immune cells, and to assess the activity of immune-related pathways. The whole process of data analysis is depicted in Figure 1.

### 2.7. Statistical Analysis

For group comparisons, we applied the Mann–Whitney *U* test (Shapiro–Wilk test, *P* < 0.05) for nonnormally distributed data and Student's *t* test for normally distributed data. All analyses were performed with *R* version 4.0.3 (https://www.r-project.org/).

## 3. Results

### 3.1. Consensus Clustering of 14 RAS-Related Prognostic Genes Identified Two Clusters of Osteosarcoma

**Figure 2:**
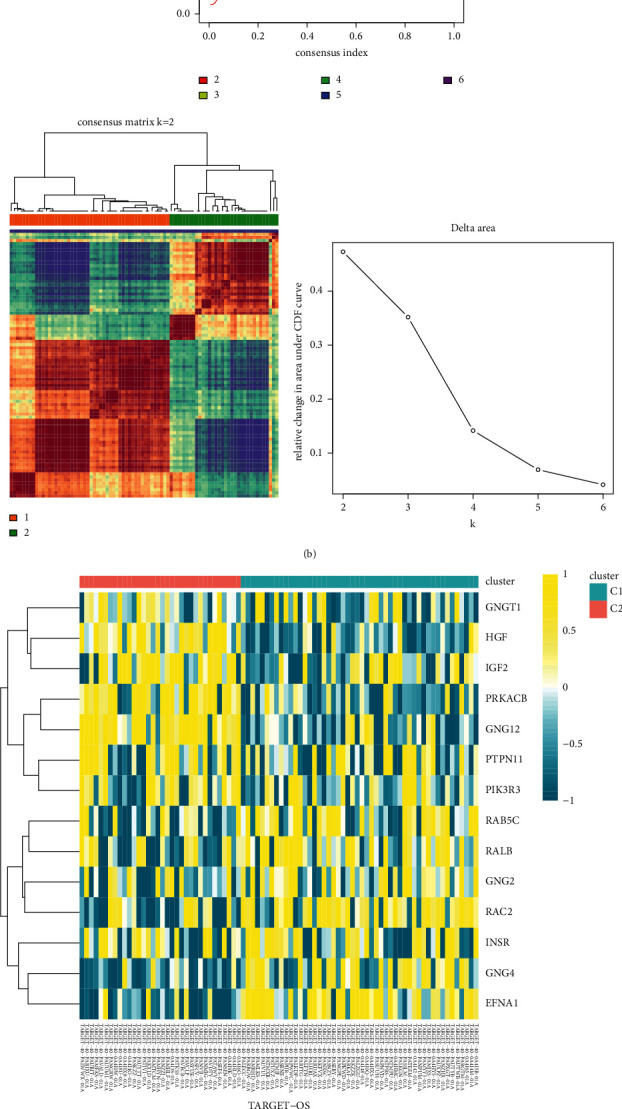
Identification of RAS-related prognostic genes. (a) Venn diagram showing the 14 intersection RAS-related prognostic genes. (b) The optimal number of clusters (*K* = 2) determined from cumulative distribution function (CDF) curves, and the classification effect is the best. (c) Heatmap of the expression of 14 RAS-related prognostic genes in the two clusters. (d) PCA confirming that osteosarcoma can be clearly separated into two clusters based on the expression of 14 RAS-related prognostic genes. (e) Kaplan–Meier curves for survival prediction of patients in the two clusters.

A total of 84 osteosarcoma patients from TARGET cohort were included in the analysis. First, we performed prognostic analysis for all genes, and 784 genes were selected (*P* < 0.05). Subsequently, 14 of 232 RAS-related genes were related to patient survival (Figure 2(a)). Then, the consensus clustering approach was conducted to divide the osteosarcoma patients into subgroups based on 14 RAS-related prognostic genes generated from univariable Cox analysis (Figure 2(a)). The optimal clustering stability was identified when *K* = 2 (Figure 2(b)). 51 patients were clustered into *C*1 and 42 patients were clustered into *C*2. The expression level of the 14 prognostic genes in the two subtypes was visualized through the heatmap (Figure 2(c)), and obvious expression difference was found between *C*1 and *C*2. Principal component analysis confirmed that osteosarcoma patients could be clearly separated into two clusters based on the 14 RAS-related prognostic genes (Figure 2(d)). Moreover, patients in the *C*2 enjoyed better overall survival than patients in the *C*1 (*P*=0.002; Figure 2(e)). These results demonstrated that the 14 RAS-related prognostic genes classify the osteosarcoma patients into two molecular subtypes with different overall survival. Consensus clustering methods were then performed to classify patients with osteosarcoma into subgroups based on the 14 RAS-related prognosis genes generated by univariate Cox analysis (Figure 2(a)). The best cluster stability was determined when *K* = 2 (Figure 2(b)). 51 patients were categorized into group *C*1 and 42 patients were categorized into group *C*2. The expression levels of the 14 prognostic genes in the two subtypes were visualized with a heat map (Figure 2(c)), and a significant expression difference was found between *C*1 and *C*2. Principal component analysis confirmed that patients with osteosarcoma could be clearly divided into two clusters based on the 14 RAS-related prognostic genes (Figure 2(d)). In addition, patients with *C*2 had better overall survival than those with *C*1 (*P*=0.002; Figure 2(e)). These results showed that 14 RAS-associated prognostic genes divided osteosarcoma patients into two molecular subtypes with different overall survival.

### 3.2. DEG and Functional Analyses

**Figure 3:**
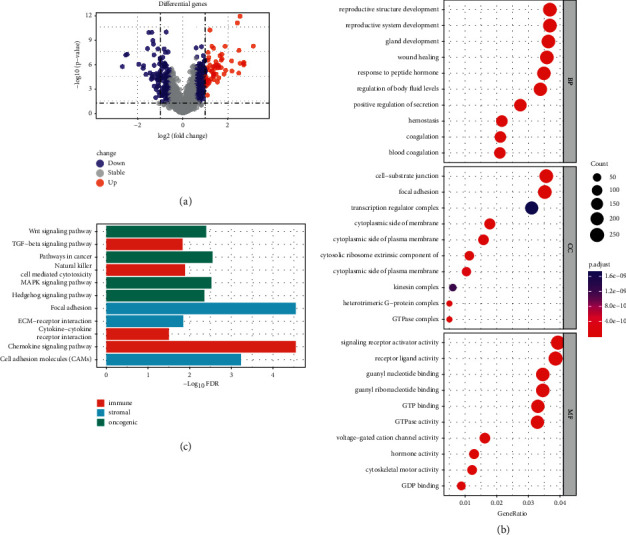
Differentially expressed gene (DEG) analysis and functional analysis. (a) Volcano plot showing the DEGs between the two subgroups. (b) Circle plot visualizing the biological processes enriched by gene ontology (GO) analysis. (c) The KEGG pathways associated with immune, stromal, and oncogenic signatures highly enriched in *C*1 versus *C*2 identified by GSEA.

DEGs were identified between the two clusters, and functional analysis was performed to explore potential signaling mechanisms. A total of 198 DEGs were identified, of which 239 genes were downregulated and 126 genes were upregulated in *C*1 compared to *C*2 (Figure 3(a)). The GO enrichment analysis indicated that DEGs were concentrated in immune-related and other biological processes, which included focal adhesion, GTP binding, cytoskeletal motor activity, and cytosolic-matrix junctions (Figure 3(b)).

Furthermore, genes differentially expressed in the two clusters were identified and subsequently analyzed for their biological processes using the KEGG pathway to annotate their functions. The results showed that *C*1-enriched pathways are mainly involved in immune response, stromal properties, and tumorigenesis (Figure 3(c)). Immune-related pathways included natural killer cell-mediated cytotoxicity, cytokine-cytokine receptor interactions, chemokine signaling, and TGF-*β* signaling. Pathways associated with stromal features include ECM receptor interactions, focal adhesion, and cell adhesion molecules. Oncogenic pathways included Hedgehog signaling, Notch signaling, MAPK signaling, Wnt signaling, and cancer pathways. The above results suggest that RAS signaling is clearly involved in cancer development, especially by affecting immune-related functions.

### 3.3. Development of a Prognostic Gene Model

**Figure 4:**
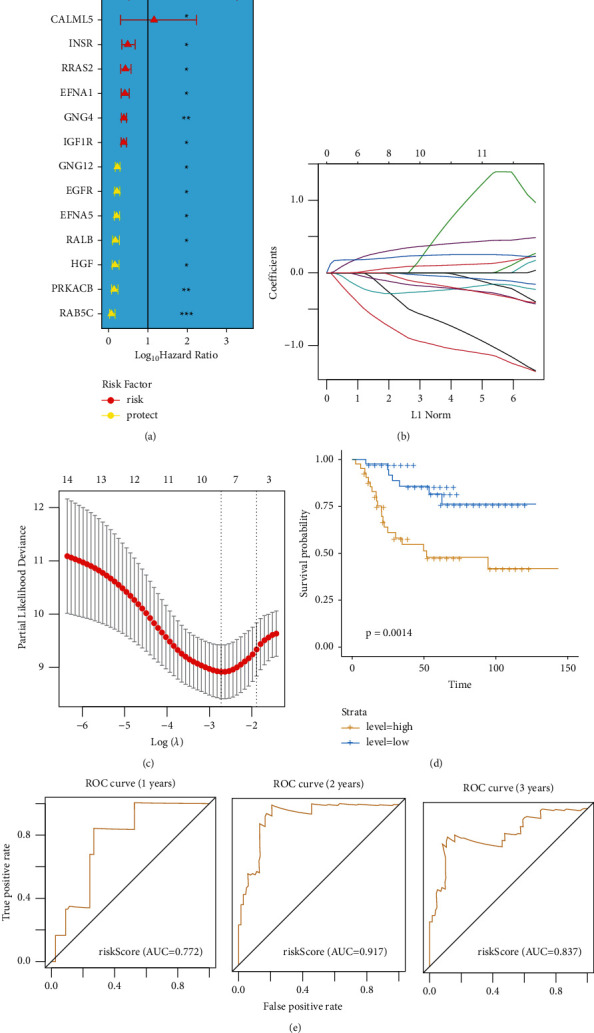
Construction of risk signature. (a) Univariate Cox regression analysis of OS for each pyroptosis-related gene, and 14 genes with *P* < 0.05. (b) LASSO regression of the 5 OS-related genes. (c) Cross-validation for tuning the parameter selection in the LASSO regression. (d) Kaplan–Meier curves for the OS of patients in the high and low-risk groups. (e) ROC curves demonstrating the predictive efficiency of the risk score.^*∗*^*P* value less than 0.05 is statistically significant.

In total, 84 OS samples were compared with appropriate patients with complete survival data for matching. A univariate Cox regression analysis was used as a primary screen for survival-associated genes. The 14 genes (CALML3, CALML5, INSR, RRAS2, EFNA1, GNG4, IGF1R, GNG12, EGFR, EFNA5, RALB, HGF, PRKACB, and RAB5C) that met the criteria of *P* < 0.05 were retained for further analysis, and among them, 7 genes (CALML3, CALML5, INSR, RRAS2, EFNA1, GNG4, and IGF1R) were associated with increased risk with HRs >1, while the other 7 genes (GNG12, EGFR, EFNA5, RALB, HGF, PRKACB, and RAB5C) were protective genes with HRs <1 (Figure 4(a)).

Five genetic features were constructed based on the optimal *λ* values by Cox regression analysis with the least absolute shrinkage and selection operator (LASSO) (Figures 4(b) and 4(c)). Risk scores were calculated as follows: risk score = (0.139 *∗* GNG4 index) + (−1.628 *∗* RAB5C index) + (−0.931 *∗* PRKACB index) + (0.447 *∗* EFNA5 index) + (0.288 *∗* EFNA1 index). Based on the median calculated by the risk score formula, 84 patients were equally divided into low and high-risk subgroups (Figure 4(d)). A significant difference in OS time was detected between the low and high-risk groups (*P* < 0.001, Figure 4(d)). Using time-dependent receiver operating characteristic (ROC) analysis to assess the sensitivity and specificity of the prognostic model, we found that the area under the ROC curve (AUC) was 0.772 at 1 year, 0.917 at 2 years, and 0.837 at 3 years of survival (Figure 4(e)).

### 3.4. Independent Prognostic Value of the Risk Model

**Figure 5:**
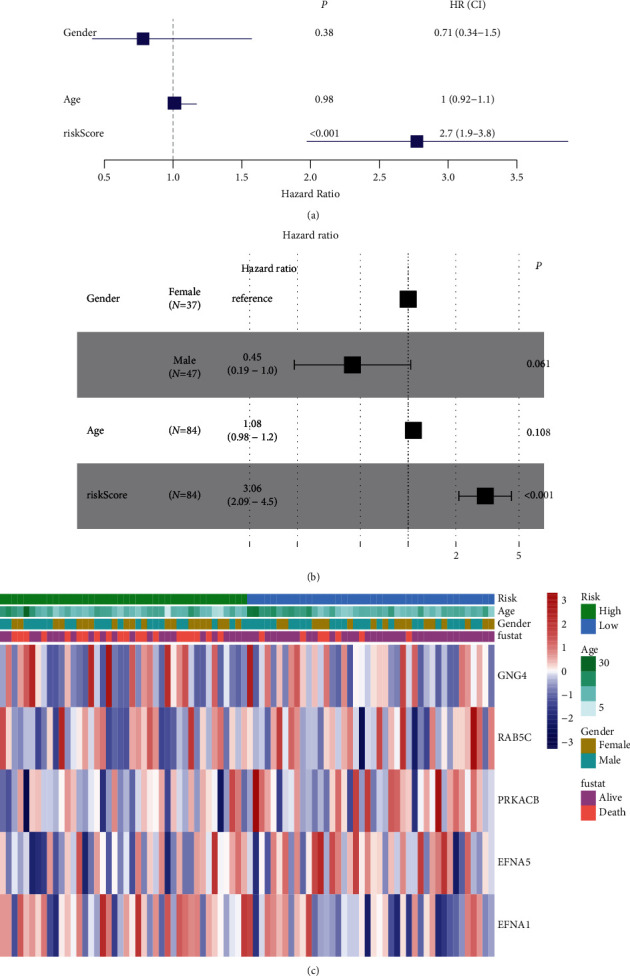
Univariate and multivariate Cox regression analyses for the risk score. (a) Univariate analysis. (b) Multivariate analysis. (c) Heatmap (green, low expression; red, high expression) for the connections between clinicopathologic features and the risk groups (^*∗*^*P* < 0.05).

We used both univariate and multivariate Cox regression analyses to evaluate whether the risk score derived from the gene profile model could be used as an independent prognostic factor. Univariate Cox regression analysis showed that risk score was an independent predictor of poor survival (HR = 2.7, 95% CI: 1.9–3.8; Figure 5(a)). Multivariate analysis also showed that risk score was a prognostic factor for OS *i* patients after adjusting for other confounders (HR = 3.06, 95% CI: 2.09–4.5; Figure 5(b)). Furthermore, a heat map of clinical characteristics was generated (Figure 5(c)), which revealed a different distribution of patients' age and survival status between low and high-risk subgroups (*P* < 0.05).

### 3.5. Comparison of the Immune Activity between Subgroups

**Figure 6:**
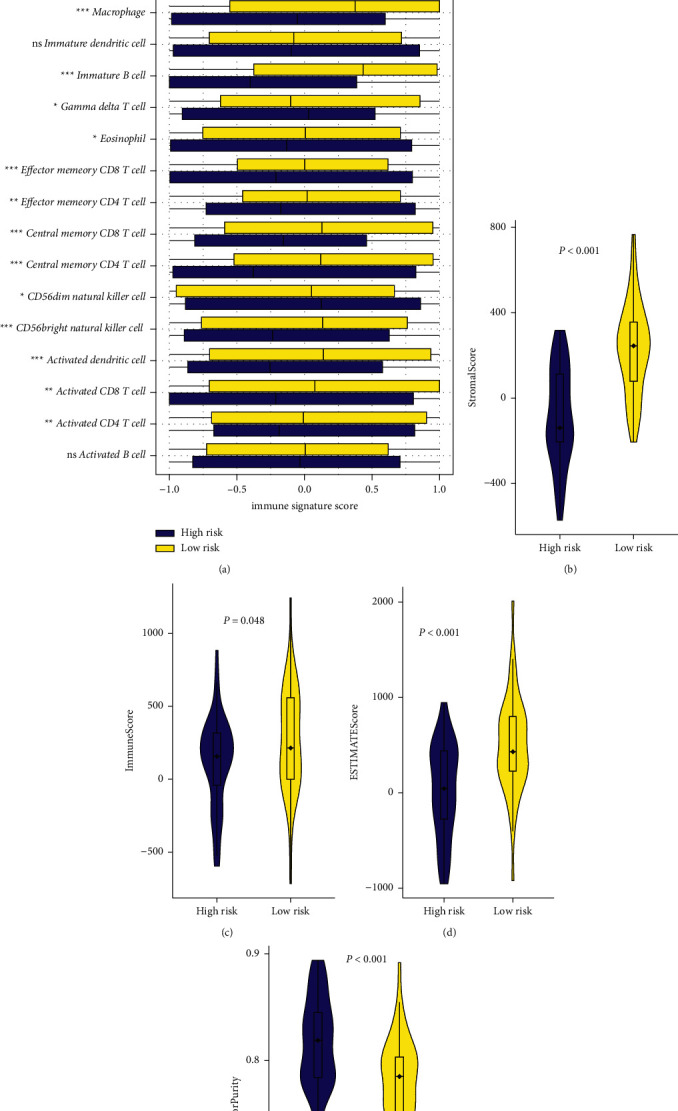
Immune analyses in the two subgroups. (a) Stromal score, (b) immune score, (c) ESTIMATE score, and (d) tumor purity calculated by the ESTIMATE algorithm. (e) Comparison of the enriching level of 29 immune-related cells evaluated by the ssGSEA algorithm, between low (yellow box) and high-risk (blue box) group. ^*∗*^*P* < 0.05; ^*∗∗*^*P* < 0.01; and ^*∗∗∗*^*P* < 0.001.

At first, the level of enrichment of 24 immune features representing the total immune activity in OS was quantified by ssGSEA. It was found that the immune cell distribution was significantly higher in the low-risk group than in the high-risk group (*P* < 0.05, Figure 6(a)). In addition, the ESTIMATE algorithm was performed to assess the time of both the groups, and the results showed that the low-risk group had significantly higher stromal score (*P* < 0.001, Figure 6(b)), immune score (*P*=0.048, Figure 6(c)), ESTIMATE score (*P* < 0.001, Figure 6(d)), and significantly lower tumor purity compared to the high-risk group (Figure 6(e)). Those results indicate that the constructed risk model has strong potential in predicting the prognosis of patients with osteosarcoma and is significantly associated with the time of osteosarcoma.

### 3.6. Risk-Based Modeling for Functional Analysis

**Figure 7:**
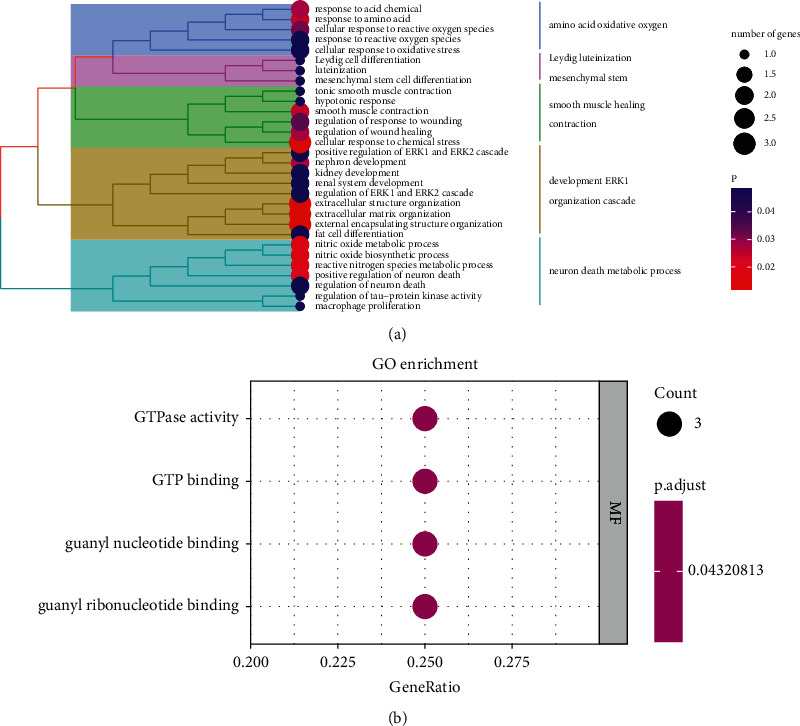
Functional analysis based on the DEGs between the two risk groups. (a) Gene ontology (GO) highly enriched in the high-risk group. (b) Gene ontology (GO) highly enriched in the low-risk group.

As further effort to explore differences in gene function and pathways between subgroups classified according to the risk model, we extracted DEGs using the *R* package “Limma” using the criteria of *P* < 0.05 and |log2FC| ≥0.585. A total of 19 DEGs were identified between the low and high-risk groups. Of these, 13 genes were upregulated in the high-risk group, while the other 6 genes were downregulated. Gene ontology (GO) enrichment analysis was then performed based on these DEGs. The results indicated that the DEGs upregulated in the high-risk group was mainly correlated with the neuron death metabolic process, organization cascade, development of ERK1, contraction, smooth muscle healing, mesenchymal stem, Leydig luteinization, and amino acid oxidative oxygen (Figure 7(a)), while the DEGs upregulated in the low-risk group was mainly correlated with GTPase activity, GTP binding, guanyl nucleotide binding, and guanyl ribonucleotide binding (Figure 7(b)).

## 4. Discussion

Osteosarcoma (OS) arises from malignant mesenchymal stem cells that generate the osteoid or immature bone [21]. For the past three decades, a combination of surgical resection and systemic chemotherapy has been the standard of care for OS, but little progress has been made since then. As the understanding of molecular mechanisms and pathways of OS has advanced, there is evidence that we are on the cusp of a paradigm shift.

Several large RNA-seq studies have revealed frequent RAS pathway abnormalities in malignancies, and the biology of the RAS pathway has been extensively reviewed [11, 22]. In view of that, RAS pathway inhibitors are available in the clinics for treating diverse cancer [12, 23]. RAN has been shown to play an important role in tumorigenesis and development through bioinformatic approaches or experiments. For example, Feng and his colleagues analyzed the development of ovarian cancer in tumors through a series of bioinformatics and identified validated biomarkers [24, 25]. Nevertheless, a comprehensive analysis of the RAS pathway in OS is still scarce and the underlying mechanisms are not fully elucidated. In our study, we attempted to construct a prognostic model by defining disease subclassifications based on RAS-related genes. First, we identified two distinct subtypes based on 14 RAS-associated prognostic genes in the TARGET osteosarcoma cohort. Compared to patients with cluster *C*2, patients with cluster *C*1 had worse overall survival. Subsequently, KEGG pathways analysis revealed that pathways enriched in *C*1 were mostly associated with immune-related responses, stromal signatures, and oncogenesis, indicating that the RAS signaling clusters were highly correlated with osteosarcoma progression. Furthermore, a 5-gene signature including *GNG4*, *RAB5C*, *PRKACB*, *EFNA5*, and *EFNA1* was established using LASSO analysis. Then, the robust predication of this RAS-related gene prognostic model was proved by ROC analysis. The aforementioned results displayed that identification of the RAS-related genes signature may provide new insight into treatment and clinical outcome predication.

RAS signaling pathway oncogenesis can be suppressed directly by targeting upstream and downstream molecules or RAS proteins directly [26]. The RAS proteins cycling between inactive and active states act as molecular switches in cell growth and differentiation [27]. The member of RAS superfamily of small GTPases has an essential function in tumor migration and invasion in osteosarcoma [28]. GO enrichment analysis demonstrated that the DEGs between RAS-related clusters were different biological processes, including GTP/GDP binding, focal adhesion, cytoskeletal motor activity, and cell-substrate junction. GTP/GDP binding regulates various cellular responses and is associated with tumor progression [29]. Thus, it may be pharmaceutically targeted in osteosarcoma treatment according to the above analysis.

RAS protein is activated by upstream receptors, such as members of the epidermal growth factor receptor (EGFR) family [30–32]. EGFR is a type of receptor tyrosine kinase (RTK) protein and located on the surface of solid tumors [33]. In this study, the EGFR expression level of osteosarcoma samples was verified by immunohistochemical staining. Currently, clinical trials are being conducted to examine the effectiveness of tyrosine kinase inhibitors (TKI) in the advanced osteosarcoma treatment. Multiple TKIs including sorafenib, regorafenib, and cabozantinib have proved activity and efficacy in patients with relapsed or metastatic osteosarcoma [13, 33, 34].

After activation of RAS proteins, downstream effector pathways are triggered, including RAS-RAF-MEK-ERK and RAS-PI3K-AKT-mTOR [35]. Suppression of the ERK signaling pathway inhibits osteosarcoma invasion and promotes apoptosis in osteosarcoma [36]. There is increasing evidence that components of the PI3K/AKT signaling pathway are often overactivated and are importantly associated with pulmonary metastasis in osteosarcoma [37]. Furthermore, osteosarcoma cell lines respond to therapeutic inhibition of the PI3K/mTOR pathway both in vitro and in vivo [38]. Therefore, targeting RAS downstream effector pathways may have a potential role in the treatment of osteosarcoma, with several agents showing promise as antimetastatic agents [39–42].

RAS pathway has not yet been fully understood in terms of its effect on immune infiltration and TME in osteosarcoma. Osteosarcoma patients with lung metastases had significantly increased expression level of exosomal PD-L1 than those with localized disease, and preclinical studies suggested that osteosarcoma may be susceptible to immunotherapy [43, 44]. Besides, it is worth mentioning that macrophages and other types of immune cells should receive more attention in osteosarcoma rather than *T* cells [45]. In this study, we discovered that the characteristics of the TME and the relative abundance of 24 immune infiltration cells differed significantly between two risk groups. Those in the RAS low-risk group had a better prognosis, showing a higher infiltration of macrophages and CD8+-infiltrating lymphocytes. In addition, the RAS low-risk group had lower tumor purity but higher ESTIMATE scores. These results suggest that the established RAS-related osteosarcoma risk model is significantly associated with TME and immune infiltration and will provide new ideas for immunotherapy. There are several limitations in our study. First, the RAS-related prognosis model based on TARGET database was not validated externally due to the scarcity of osteosarcoma data. Second, the osteosarcoma samples of the risk model were retrospectively extracted from the public database, and there was existing inherent case selection bias in this study. Finally, additional in vitro and in vivo experiments are needed to validate RAS-associated prognostic genes in osteosarcoma.

## 5. Conclusion

In conclusion, our comprehensive analysis of the RAS pathway in osteosarcoma revealed its significant value in prognosis. Besides, RAS-related gene subtypes were involved in different molecular pathways related to tumor progression and immune infiltration. These findings spotlight the important clinical implications of RAS, and further research is required to investigate the therapeutic value about RAS-related gene in osteosarcoma.

## Data Availability

The datasets analyzed during the current study are available in the TCGA repository, https://www.cancergeno.me.nih.gov/.
